# Body measurement changes in adults with pectus excavatum after the Nuss procedure: a study of 272 patients

**DOI:** 10.1186/s13019-024-02573-6

**Published:** 2024-02-06

**Authors:** Nay Htut, I-Shiang Tzeng, Yu-Jiun Fan, Yeung-Leung Cheng

**Affiliations:** 1 Division of Thoracic Surgery, Department of Surgery, Taipei Tzu Chi Hospital, Buddhist Tzu Chi Medical Foundation, New Taipei City, Taiwan; 2 Department of Research, Taipei Tzu Chi Hospital, Buddhist Tzu Chi Medical Foundation, New Taipei City, Taiwan; 3 Department of Statistics, National Taipei University, New Taipei City, Taiwan; 4 School of Medicine, Tzu Chi University, Hualien, Taiwan

**Keywords:** Pectus Excavatum, Nuss procedure, Body measurement, Body development, Physical growth, Body weight, Body height

## Abstract

**Background:**

Pectus excavatum (PE) is the most common congenital abnormality of the chest wall. Most patients with PE have slim bodies. Some studies have been conducted on the physical growth of children and adolescents who underwent the Nuss procedure. This study aimed to evaluate body measurement changes in adult patients with PE after the Nuss procedure.

**Methods:**

A total of 272 adult PE patients, who underwent the Nuss procedure and pectus bars removal from August 2014 to December 2020, were evaluated retrospectively. Body measurement [body height (BH), body weight (BW), and body mass index (BMI)] of the patients were collected before Nuss repair and after bar removal. We used the interquartile range (IQR) to identify and exclude outliers. Associations between changes in body measurement and clinical and radiological features were evaluated.

**Results:**

The BH, BW and BMI showed significantly increased after pectus bar removal, compared to pre-Nuss procedure parameters (BH 173.8 ± 5.9 cm vs. 173.9 ± 5.9 cm, *P* < 0.001; BW 60.3 ± 8.1 kg vs. 61.1 ± 8.8 kg, *P* = 0.005; BMI 19.9 ± 2.2 kg/m2 vs. 20.1 ± 2.4 kg/m2, *P* = 0.02). The same result were observed in the male subgroup, the HI ≥ 4 group and the male subgroup within the HI ≥ 4 group.

**Conclusions:**

The BH, BW and BMI were significantly increased after completing surgical correction of PE using the Nuss procedure, particularly in young males and patients with more pronounced deformities.

**Supplementary Information:**

The online version contains supplementary material available at 10.1186/s13019-024-02573-6.

## Background

Pectus excavatum (PE), or funnel chest, is the most frequent chest wall deformity and accounts for the highest incidence among all congenital chest wall anomalies. PE presents with anterior chest wall depression due to dorsal deviation of the sternum and the third to seventh rib or costal cartilage [1]. PE is mainly diagnosed in the neonatal period but may become apparent with age [2]. These patients are generally characterized by thin and tall body shapes, and it is believed that more severe PE may be accompanied by an increased risk of growth restriction [1, 3].

Surgical correction is a standard treatment for PE. The Nuss procedure is one of the most common approaches to PE repair; it involves applying outward pressure to the inward sternum using a custom-contoured Nuss bar without cartilage resection [4]. As technology matures, PE correction for children and young people can also be used for adult PE correction.

Whether the body measurement of patients with PE can improve after surgery is a topic worthy of discussion. Some studies have reported improvement of body weight (BW) and body height (BH) gain in children with PE after the Nuss procedure [3, 5]. Therefore, we were intrigued about whether adult population also exhibits a similar outcome. However, there is little information about changes in the body measurement in adult population with PE who underwent the Nuss procedure [6]. This retrospective study focused on changes in the body measurement in young adults who underwent the Nuss procedure for PE.

## Methods

### Patients

Adult patients (≥ 18 years old), who underwent Nuss repair for PE followed by bar removal surgery between August 2014 and December 2020 at our hospital, were assessed retrospectively. Preoperative and post-bar-removal parameters were collected, including BW (kg), BH (cm), body mass index (BMI), sex, and Haller index (HI) [7, 8]. We compared preoperative and post-bar-removal body measurements to assess the surgical effect on the patients. The subgroup was divided by sex. The age cutoff was set at 25 years for better group balance. We used a Haller index ≥ 4 as a cutoff point for severe cases, following the precedent set by previous studies [9, 10]. The Institutional Review Board and the Ethics Committee of the Taipei Tzu-Chi Hospital agreed with the publication of this study, Taipei, Taiwan, ROC (IRB No: 09-XD-097). The Institutional Review Board waived the need for patient consent due to the study’s retrospective nature.

The major indications for surgery are based on at least two of the following conditions: (1) progressive dyspnea or chest pain, (2) restrictive ventilatory defect, (3) exercise intolerance, (4) aggravation of the deformity, (5) presence of cardiac compression, (6) HI > 3.25, and (7) mitral valve prolapse [11–13]. The correction duration was approximately 3 years, and we excluded patients with a correction duration of < 2 years or > 6 years.

### Body weight and height measurements

In the process of measuring body weight and height, we adhere to standardized procedures to ensure accuracy and consistency. These measurements are conducted at the hospital.

### Body weight measurement

For measuring BW, we employ a precise and calibrated digital weighing scale. First, we instruct patients to don lightweight clothing and avoid carrying any heavy items. Prior to use, we meticulously calibrate the scale to read zero, ensuring an accurate starting point. Patients are then positioned on the scale with their weight evenly distributed between both feet. Subsequently, we record the stabilized weight reading in kilograms.

### Body height measurement

To measure BH, we utilize a stadiometer calibrated in centimeters as the unit of measurement. Patients are asked to either stand barefoot or wear socks. They are guided to stand with their back and heels flush against the stadiometer, ensuring their feet rest flat on the floor. Their head is aligned with the Frankfort horizontal plane, maintaining a horizontal line of vision. We gently lower the measuring rod or headpiece of the stadiometer until it lightly contacts the top of the patient’s head, ensuring it remains perpendicular to the ground. For precision, we take multiple height measurements and calculate the average for an accurate height assessment.

### Operative procedures

#### Nuss procedure

A modified Nuss procedure with bilateral thoracoscopy was performed with right-to-left mediastinal dissection [14–16]. In brief, the patient was placed in the supine position, and the patient’s arms were abducted at approximately 70° relative to the body’s longitudinal axis, after general anesthesia with a single-lumen endotracheal tube. We dissected the subcutaneous and submuscular tissues and opened the pleural cavity near the highest point of the deformity. An intraoperative thoracoscope was routinely applied to watch the introducer to create a mediastinal dissection from right to left to avoid cardiac injury. One to three bars (Zimmer Biomet, Jacksonville, FL, USA) with a pre-bent to bridge shape were pulled back by connecting them to a 28-Fr chest tube via the substernal tunnel. The pectus bar was rotated, anchored, and fixed with either heavy non-absorbing sutures or a 1-mm stainless steel wire across the nearby rib at both ends of the bar. After completion of anesthesia, the patients underwent chest radiography and were closely monitored for 12–24 hours.

#### Surgical techniques for bars removal

The pectus bars were generally removed after at least 2 years of repair. This procedure was performed as previously described [15]. After general anesthesia and single-lumen intubation, the patient was placed in the supine position, and their arms were abducted. The skin was incised through previous scars, the end of the bars were exposed, and the fixation materials and bars were removed. Bleeding on the operation field and rough surfaces of the callus were checked, and hemostatic gauze was applied to prevent oozing. Finally, the incisional wound was closed, covered, and compressed using a 6-inch elastic bandage.

### Postoperative care and follow-up

Patient-controlled epidural analgesia with opioids (morphine or fentanyl) and nonsteroidal anti-inflammatory drugs (NSAID) were used for analgesia. The patients were discharged with adequate pain control using oral analgesics. Regular follow-up appointments were conducted at 2 weeks, 1 month, 3 months, and 6 months, and then once or twice annually until bar removal. After bar removal, the pain was controlled by NSAID, and patients were mostly discharged on the third postoperative day. Outpatient clinic follow-up was 2 weeks after bar removal and annually thereafter.

### Statistical analysis

Mean ± SD (standard deviation) or range were used for descriptive data and continuous variables. For continuous data, Student’s t-test was performed to compare postoperative and preoperative values. We conducted a paired t-test to assess the differences in body measurements between time points. Any body measurements that changed and fell outside the range of Q1 – 1.5 times the interquartile range (IQR) or Q3 + 1.5 times the IQR were categorized as outliers. The chi-squared or Fisher’s exact test was performed for between-group comparisons of categorical variables. Statistical significance was defined as a P-value of < 0.05. SPSS (version 24; IBM, Armonk, NY, USA) was used for statistical analyses.

## Results

The mean age of the patients receiving the Nuss procedure (pre-Nuss procedure, pre-N) was 24.9 ± 4.6 (range: 18–43) years, and the mean age after removal of the bar (post-bar removal, post-R) was 29.0 ± 4.9 (range: 22–48) years (Table 1). The mean interval of correction duration was 3.6 ± 1.2 years (range: 2.8–6.0 years). The distributions of age and sex at the time of repair are shown in Supplementary Fig. 1, Additional File 1. Most patients underwent correction using two bars (217 patients, 79.7%). The bar size ranged from 11 to 14 in. All bars were successfully removed. The mean preoperative chest X-ray HI (CXR-HI) was 4.05 ± 1.17, and 3.21 ± 0.64 in postoperative bar removal CXR-HI. Postoperative CXR-HI was significantly improved compared to preoperative CXR-HI (*P* < 0.01).

**Table 1:** Patient characteristics

Characteristics (*n* = 272)	
Age, mean ± SD	
pre-N	24.9 ± 4.6 (range: 18–43)
post-R	29.0 ± 4.9 (range: 22–48)
Male (%)	255 (93.7)
Female (%)	17 (6.2)
Body height, cm, mean ± SD	
pre-N	174 ± 6.1 (range: 152–190)
post-R	174.3 ± 6.2 (range: 152–190)
Body weight, kg, mean ± SD	
pre-N	61.3 ± 8 (range: 40–95)
post-R	62.9 ± 9.5 (range: 39.2–112.6)
BMI, mean ± SD	
pre-N	20.1 ± 2.4 (range: 13.9–29.2)
post-R	20.4 ± 2.6 (range: 14.9–35.54)
Interval of correction years, mean ± SD	3.6 ± 1.2 (range: 2.8–6.0)
Chest X-ray Haller index, mean ± SD	
pre-N	4.05 ± 1.17 (range: 2.50–11.70)
post-R	3.21 ± 0.64 (range: 2.02–6.60)
Bar numbers	
One Bar (%)	55 (20.2)
Two Bars (%)	217 (79.7)

Characteristics of patients before the Nuss procedure (pre-Nuss procedure, *pre-N*) and after bar removal (post-bar removal, *post-R*). *SD* standard deviation, *cm* centimeter, *kg* kilogram, *BMI* body mass index

After the exclusion of outliers, the changes in postoperative body measurements for sex, age, and HI are shown in Supplementary Tables 1–3, Additional File 1. BH, BW, and BMI showed significant increases post-operation compared to pre-operation measurements (*N* = 183) (BH 173.8 ± 5.9 cm vs. 173.9 ± 5.9 cm, *P* < 0.001; BW 60.3 ± 8.1 kg vs. 61.1 ± 8.8 kg, *P* = 0.005; BMI 19.9 ± 2.2 kg/m2 vs. 20.1 ± 2.4 kg/m2, *P* = 0.02) (Supplementary Table 1). Moreover, in males, BH, BW, and BMI exhibited significantly greater values post-operation than pre-operation (*N* = 171) (BH 174.2 ± 5.7 cm vs. 174.3 ± 5.7 cm, *P* < 0.001; BW 60.8 ± 7.8 kg vs. 61.6 ± 8.5 kg, *P* = 0.004; BMI 20.0 ± 2.2 kg/m2 vs. 20.2 ± 2.4 kg/m2, *P* = 0.015). However, among females, only BH showed a statistically significant change (*N* = 12) (BH 168.0 ± 6.9 cm vs. 168.2 ± 6.8 cm, *P* = 0.037).

In the older age group (≥ 25 years, *N* = 77), the only significant postoperative change observed was in BH (BH 173.1 ± 6.4 cm vs. 173.2 ± 6.4 cm, *P* = 0.004) (Supplementary Table 2). In the male subgroup (*N* = 71) of this age group, BH also showed a statistically significant change (BH 173.7 ± 6.2 cm vs. 173.8 ± 6.2 cm, *P* = 0.019). However, no significance was noticed in the female subgroup (*N* = 6). In the young age group (< 25 years, *N* = 106), postoperative BH and BW showed significant increases compared to their respective preoperative values (BH 174.2 ± 5.5 cm vs. 174.5 ± 5.5 cm, *P* < 0.001; BW 59.5 ± 6.8 kg vs. 60.3 ± 8.2 kg, *P* = 0.04). A similar trend was observed in the subgroup of young males (*N* = 100) (BH 174.5 ± 5.3 cm vs. 174.7 ± 5.3 cm, *P* < 0.001; BW 59.7 ± 6.5 kg vs. 60.7 ± 8.0 kg, *P* = 0.026) but not in the subgroup of young females (*N* = 6).

In the HI ≥ 4 group (*N* = 76) (Supplementary Table 3), postoperative BH, BW, and BMI exhibited significant increases (BH 174 ± 5.8 cm vs. 174.3 ± 5.8 cm, *P* < 0.001; BW 59.3 ± 8.5 kg vs. 60.5 ± 8.9 kg, *P* < 0.007; BMI 19.5 ± 2.3 kg/m² vs. 19.9 ± 2.4 kg/m², *P* = 0.015). Among males in the HI ≥ 4 group (*N* = 77), postoperative BH, BW, and BMI also showed significant increases (BH 174.5 ± 5.5 cm vs. 174.7 ± 5.5 cm, *P* < 0.001; BW 60 kg ± 8.2 kg vs. 61.2 ± 8.6 kg, *P* = 0.01; BMI 19.7 ± 2.3 kg/m² vs. 20 ± 2.4 kg/m², *P* = 0.022). However, there were no significant changes in females (*N* = 6) within the HI ≥ 4 group.

For patients with HI < 4 (*N* = 107) (Supplementary Table 3), only postoperative BH exhibited a significant increase (BH 173.6 ± 6.0 cm vs. 173.7 ± 6.0 cm, *P* < 0.001). Similarly, within the HI < 4 males subgroup (*N* = 101), BH also showed a significant increase (BH 174 ± 5.8 cm vs. 174.1 ± 5.8 cm, *P* < 0.001). In contrast, there were no significant changes in the HI < 4 females subgroup (*N* = 6).

## Discussion

PE is associated with connective tissue disorders, implying that it may be related to abnormal cartilage development [17]. Depression of the anterior chest wall is associated with sternal weakness and flexibility abnormality, rib overgrowth, and bony thorax development failure [1]. PE may occur with birth; it has an uneven progression, and deterioration of the deformity usually occurs during the pubertal growth spurt. The deformity is stable in adulthood, implying an obvious linkage with skeletal growth [17]. PE is often associated with several genetic syndromes that cause hyaline cartilage defects, thereby disturbing the structure of costal cartilage [18]. PE can cause restrictive lung ventilation, heart compression, and scoliosis, leading to exercise intolerance, poor endurance, chest discomfort, shortness of breath, forward-leaning posture and low self-esteem [1]. Park et al. reported that growth development which includes BH, BW and BMI are retarded in PE patients between 3 and 20 years of age, compared with those of the healthy population, and the degree of growth retardation is related to the timing of surgery and the severity of PE [3]. Kim et al. also reported that the release of cardiac compression after the Nuss procedure in children and teenagers with PE, gained adequate blood circulation and resulted in the restoration of BW and BH growth, suggesting that the Nuss procedure may have a positive effect on physical growth [5]. However, most of these reports are about children and adolescents; therefore, we conducted a retrospective study of adults with PE who underwent the Nuss procedure, to determine the effects on the body measurements in this population.

In this study, overall, postoperative changes in BH, BW, and BMI were significantly increased after the Nuss procedure and bar removal. The increase in BH was attributed to the correction of body posture after the Nuss procedure. The increase in BW may be related to cardiopulmonary benefits after the procedure [19], along with improved emotional well-being and self-esteem [20]. The absence of BW increases in patients aged 25 years and older could be due to the small sample size in this age group. And also due to small sample size in the female group, only BH showed a significant increase.

HI also called the pectus severity index, evaluates the severity of the pectus defect at the level of maximal depression, by comparing the ratio of the lateral diameter of the chest to the sternum-to-spine distance [7]. A normal chest HI is ≤ 2.5 and patients with a HI > 3.25 are suggested to be referred for surgery [10]. In this study, we found that adult males with a HI ≥ 4 can benefit from BH and BW growth after the Nuss procedure, and tend to show better resolution of symptoms with a satisfactory and improved quality of life. Based on our findings, it appears that young adult males with severe PE who undergo the Nuss procedure experience positive effects on both BH and BW growth. Additionally, an increase in BH and BW can improve underweight status in some patients, leading to a better overall health condition.

The current study has several limitations, including its retrospective single-center design and a relatively small sample size. Additionally, there is a lack of direct evidence regarding clinical pulmonary and cardiac function improvement, and very few cases have been reported in the adult female population. While we did not observe a significant change in BW in the older male and female subgroups, it is important to note that significant changes may become apparent with a larger sample size. Therefore, more comprehensive studies with larger sample sizes are necessary to better understand the effects of surgical intervention on the body measurements and overall health outcomes in adult population with pectus excavatum.

## Conclusions

This study showed that adult patients with PE who underwent the Nuss procedure, experienced a significant increase in BH, BW and BMI after removal of the pectus bar, and this was particularly obvious in young males and those with more severe deformities.

### Electronic supplementary material

Below is the link to the electronic supplementary material.

Supplementary Material 1

## Data Availability

All data generated or analyzed during this study are included in this published article; further inquiries can be directed to the corresponding author.

## Abbreviations

BW: Body weight
CXR: HI-Chest X-ray Haller index
HI: Haller index
PE: Pectus excavatum
BMI: Body mass index
