# Supplementary material for: Body measurement changes in adults with pectus excavatum after the Nuss procedure: a study of 272 patients
Source: J Cardiothorac Surg. 2024 Feb 6;19:65. doi: 10.1186/s13019-024-02573-6 (PMC10845464; doi:10.1186/s13019-024-02573-6)
Supplement: Supplementary file 1 — Supplementary Material 1 [file 13019_2024_2573_MOESM1_ESM.docx]

Supplementary Figure 1. Patients distribution by age and sex at the time of repair.


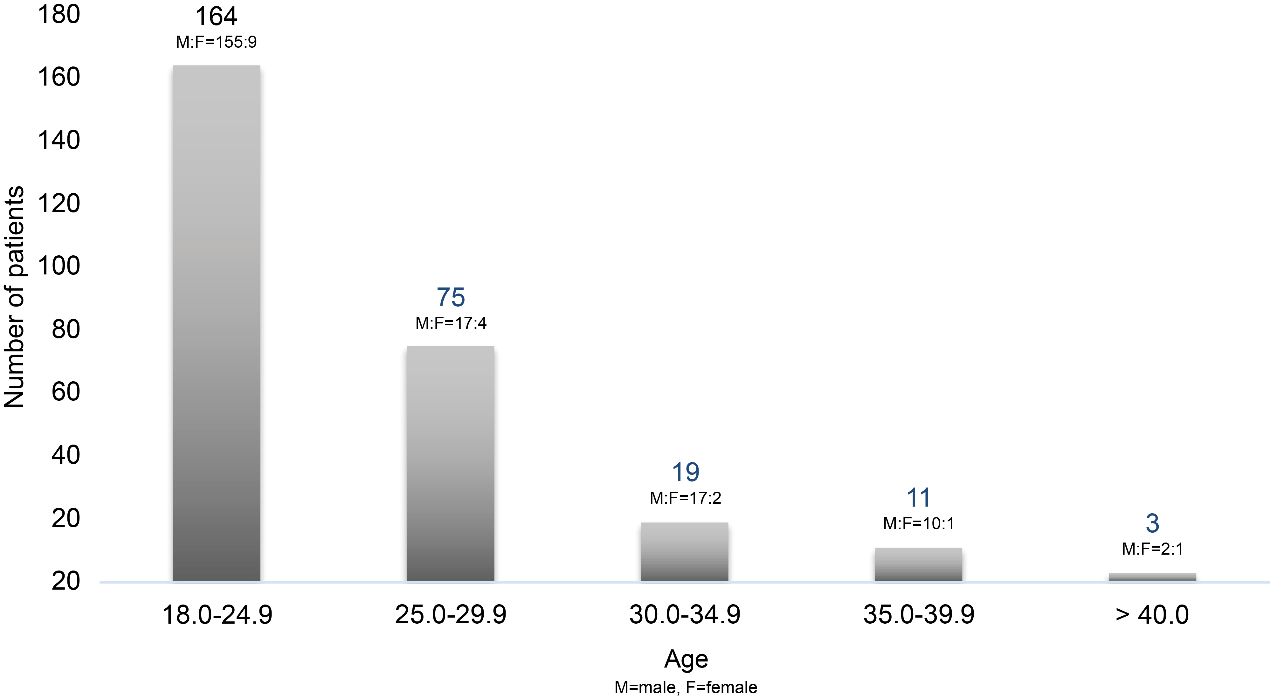


Number of patients of each sex stratified by age range before the Nuss procedure.

Supplementary Table 1. Comparisons of body measurements with respect to sex.

|  | pre-N | post-R | *P-value* |
| --- | --- | --- | --- |
| Total (n = 183) |  |  |  |
| Body height, cm, mean ± SD | 173.8 ± 5.9 | 173.9 ± 5.9 | <0.001** |
| Body weight, kg, mean ± SD | 60.3 ± 8.1 | 61.1 ± 8.8 | 0.005* |
| BMI, mean ± SD | 19.9 ± 2.2 | 20.1 ± 2.4 | 0.02* |
| Male (n = 171) |  |  |  |
| Body height, cm, mean ± SD | 174.2 ± 5.7 | 174.3 ± 5.7 | <0.001** |
| Body weight, kg, mean ± SD | 60.8 ± 7.8 | 61.6 ± 8.5 | 0.004* |
| BMI, mean ± SD | 20.0 ± 2.2 | 20.2 ± 2.4 | 0.015* |
| Female (n = 12) |  |  |  |
| Body height, cm, mean ± SD | 168.0 ± 6.9 | 168.2 ± 6.8 | 0.037* |
| Body weight, kg, mean ± SD | 53.24 ± 10.2 | 53.25 ± 9.1 | 0.989 |
| BMI, mean ± SD | 18.76 ± 2.9 | 18.73 ± 2.5 | 0.947 |

Comparisons of body measurements with respect to sex before the Nuss procedure (pre-Nuss procedure, pre-N) and after bar removal (post-bar removal, post-R). *SD* standard deviation, *cm* centimeter, *kg* kilogram, *BMI* body mass index

Supplementary Table 2. Comparisons of body measurements with respect to age.

|  | pre-N | post-R | *P-value* |
| --- | --- | --- | --- |
| ≥25 years (n = 77) |  |  |  |
| Body height, cm, mean ± SD | 173.1 ± 6.4 | 173.2 ± 6.4 | 0.004* |
| Body weight, kg, mean ± SD | 61.3 ± 9.6 | 62.17 ± 9.4 | 0.06 |
| BMI, mean ± SD | 20.4 ± 2.5 | 20.6 ± 2.3 | 0.103 |
| <25 years (n = 106) |  |  |  |
| Body height, cm, mean ± SD | 174.2 ± 5.5 | 174.5 ± 5.5 | <0.001** |
| Body weight, kg, mean ± SD | 59.5 ± 6.8 | 60.3 ± 8.2 | 0.04* |
| BMI, mean ± SD | 19.5 ± 2.0 | 19.8 ± 2.5 | 0.096 |
| ≥25 years |  |  |  |
| Male (n = 71) |  |  |  |
| Body height, cm, mean ± SD | 173.7 ± 6.2 | 173.8 ± 6.2 | 0.019* |
| Body weight, kg, mean ± SD | 62.2 ± 9.1 | 63.0 ± 9.0 | 0.069 |
| BMI, mean ± SD | 20.5 ± 2.4 | 20.7 ± 2.3 | 0.109 |
| Female (n = 6) |  |  |  |
| Body height, cm, mean ± SD | 165.6 ± 4.5 | 166 ± 4.3 | 0.066 |
| Body weight, kg, mean ± SD | 50.9 ± 10.5 | 52 ± 8.4 | 0.647 |
| BMI, mean ± SD | 18.5 ± 3.5 | 18.8 ± 2.7 | 0.719 |
| <25 years |  |  |  |
| Male (n = 100) |  |  |  |
| Body height, cm, mean ± SD | 174.5 ± 5.3 | 174.7 ± 5.3 | <0.001** |
| Body weight, kg, mean ± SD | 59.7 ± 6.5 | 60.7 ± 8.0 | 0.026* |
| BMI, mean ± SD | 19.6 ± 1.9 | 19.8 ± 2.4 | 0.066 |
| Female (n = 6) |  |  |  |
| Body height, cm, mean ± SD | 170.5 ± 8.5 | 170.5 ± 8.4 | 0.363 |
| Body weight, kg, mean ± SD | 55.5 ± 10.3 | 54.4 ± 10.5 | 0.307 |
| BMI, mean ± SD | 18.9 ± 2.4 | 18.6 ± 2.6 | 0.270 |

Comparisons of body measurements with respect to age before the Nuss procedure (pre-Nuss procedure, pre-N) and after bar removal (post-bar removal, post-R). *BMI* body mass index; *SD* standard deviation

Supplementary Table 3. Comparison of body measurements with respect to the severity of pectus deformity.

|  | pre-N | post-R | *P-value* |
| --- | --- | --- | --- |
| HI ≥ 4 (n = 76) |  |  |  |
| Body height, cm, mean ± SD | 174 ± 5.8 | 174.3 ± 5.8 | <0.001** |
| Body weight, kg, mean ± SD | 59.3 ± 8.5 | 60.5 ± 8.9 | 0.007* |
| BMI, mean ± SD | 19.5 ± 2.3 | 19.9 ± 2.4 | 0.015* |
| HI < 4 (n =107) |  |  |  |
| Body height, cm, mean ± SD | 173.6 ± 6.0 | 173.7 ± 6.0 | <0.001** |
| Body weight, kg, mean ± SD | 60.9 ± 7.8 | 61.5 ± 8.7 | 0.173 |
| BMI, mean ± SD | 20.2 ± 2.1 | 20.3 ± 2.4 | 0.315 |
| HI ≥ 4 |  |  |  |
| Male (n = 70) |  |  |  |
| Body height, cm, mean ± SD | 174.5 ± 5.5 | 174.7 ± 5.5 | <0.001** |
| Body weight, kg, mean ± SD | 60 ± 8.2 | 61.2 ± 8.6 | 0.01* |
| BMI, mean ± SD | 19.7 ± 2.3 | 20 ± 2.4 | 0.022* |
| Female (n = 6) |  |  |  |
| Body height, cm, mean ± SD | 169.3 ± 7.6 | 169.5 ± 7.5 | 0.102 |
| Body weight, kg, mean ± SD | 51.1 ± 9.0 | 52.5 ± 9.2 | 0.470 |
| BMI, mean ± SD | 17.7 ± 1.7 | 18.2 ± 2.5 | 0.454 |
| HI < 4 |  |  |  |
| Male (n = 101) |  |  |  |
| Body height, cm, mean ± SD | 174 ± 5.8 | 174.1 ± 5.8 | <0.001** |
| Body weight, kg, mean ± SD | 61.3 ± 7.5 | 61.9 ± 8.5 | 0.110 |
| BMI, mean ± SD | 20.2 ± 2.1 | 20.4 ± 2.4 | 0.196 |
| Female (n = 6) |  |  |  |
| Body height, cm, mean ± SD | 166.8 ± 6.7 | 167 ± 6.6 | 0.203 |
| Body weight, kg, mean ± SD | 55.3 ± 11.7 | 53.9 ± 9.9 | 0.355 |
| BMI, mean ± SD | 19.7 ± 3.5 | 19.2 ± 2.7 | 0.305 |

Comparison of body measurements before the Nuss procedure (pre-Nuss procedure, pre-N) and after bar removal (post-bar removal, post-R) with respect to the severity of pectus deformity. *BMI* body mass index; *SD* standard deviation; *HI* Haller index
